# In vivo hepatogenic capacity and therapeutic potential of stem cells from human exfoliated deciduous teeth in liver fibrosis in mice

**DOI:** 10.1186/s13287-015-0154-6

**Published:** 2015-09-10

**Authors:** Takayoshi Yamaza, Fatima Safira Alatas, Ratih Yuniartha, Haruyoshi Yamaza, Junko K. Fujiyoshi, Yusuke Yanagi, Koichiro Yoshimaru, Makoto Hayashida, Toshiharu Matsuura, Reona Aijima, Kenji Ihara, Shouichi Ohga, Songtao Shi, Kazuaki Nonaka, Tomoaki Taguchi

**Affiliations:** Department of Molecular Cell Biology and Oral Anatomy, Kyushu University Graduate School of Dental Science, 3-1-1 Maidashi, Higashi-ku, Fukuoka 812-8582 Japan; Department of Pediatric Surgery, Kyushu University Graduate School of Medical Sciences, 3-1-1 Maidashi, Higashi-ku, Fukuoka 812-8582 Japan; Department of Pediatric Dentistry, Kyushu University Graduate School of Dental Science, 3-1-1 Maidashi, Higashi-ku, Fukuoka 812-8582 Japan; Department of Pediatrics, Kyushu University Graduate School of Medical Sciences, 3-1-1 Maidashi, Higashi-ku, Fukuoka 812-8582 Japan; Department of Pediatrics, Faculty of Medicine, Oita University, 1-1 Idaigaoka, Hazama-cho, Yuhuin 879-5593 Japan; Department of Pediatrics, Faculty of Medicine and Health Sciences, Yamaguchi University, 1-1-1 Minami Ogushi, Ube, 755-8505 Japan; Department Anatomy and Cell Biology, School of Dental Medicine, University of Pennsylvania, 240 South 40th Street, Philadelphia, PA 19104-6030 USA

## Abstract

**Introduction:**

Liver transplantation is a gold standard treatment for intractable liver diseases. Because of the shortage of donor organs, alternative therapies have been required. Due to their potential to differentiate into a variety of mature cells, stem cells are considered feasible cell sources for liver regeneration. Stem cells from human exfoliated deciduous teeth (SHED) exhibit hepatogenic capability *in vitro*. In this study, we investigated their *in vivo* capabilities of homing and hepatocyte differentiation and therapeutic efficacy for liver disorders in carbon tetrachloride (CCl_4_)-induced liver fibrosis model mice.

**Methods:**

We transplanted SHED into CCl_4_-induced liver fibrosis model mice through the spleen, and analyzed the *in vivo* homing and therapeutic effects by optical, biochemical, histological, immunological and molecular biological assays. We then sorted human leukocyte antigen-ABC (HLA-ABC)-positive cells from primary CCl4-damaged recipient livers, and analyzed their fusogenicity and hepatic characteristics by flow cytometric, genomic DNA, hepatocyte-specific gene assays. Furthermore, we examined the treatment effects of HLA-positive cells to a hepatic dysfunction by a secondary transplantation into CCl_4_-treated mice.

**Results:**

Transplanted SHED homed to recipient livers, and expressed HLA-ABC, human hepatocyte specific antigen hepatocyte paraffin 1 and human albumin. SHED transplantation markedly recovered liver dysfunction and led to anti-fibrotic and anti-inflammatory effects in the recipient livers. SHED-derived HLA-ABC-positive cells that were sorted from the primary recipient liver tissues with CCl_4_ damage did not fuse with the host mouse liver cells. Sorted HLA-positive cells not only expressed human hepatocyte-specific genes including albumin, cytochrome P450 1A1, fumarylacetoacetase, tyrosine aminotransferase, uridine 5′-diphospho-glucuronosyltransferase, transferrin and transthyretin, but also secreted human albumin, urea and blood urea nitrogen. Furthermore, SHED-derived HLA-ABC-positive cells were secondary transplanted into CCl_4_-treated mice. The donor cells homed into secondary recipient livers, and expressed hepatocyte paraffin 1 and human albumin, as well as HLA-ABC. The secondary transplantation recovered a liver dysfunction in secondary recipients.

**Conclusions:**

This study indicates that transplanted SHED improve hepatic dysfunction and directly transform into hepatocytes without cell fusion in CCl_4_-treated mice, suggesting that SHED may provide a feasible cell source for liver regeneration.

**Electronic supplementary material:**

The online version of this article (doi:10.1186/s13287-015-0154-6) contains supplementary material, which is available to authorized users.

## Introduction

Hepatic fibrosis is a severe chronic condition that occurs as a result of various congenital and acquired hepatic disorders, including viral, drug-induced, cholestatic, metabolic, and autoimmune diseases. Cirrhosis, the most advanced stage of hepatic fibrosis, usually progresses to hepatocellular carcinoma, resulting in liver failure without the liver’s usual self-regenerative capability. Unfortunately, current pharmaceutical and immunological treatments are unable to cure patients with hepatic fibrosis and/or cirrhosis. Liver transplantation is therefore the only treatment with clinical success. However, few patients benefit from organ grafting because of high medical expenses, the long-term wait for a donor liver, organ rejection, and complications [1]. Hepatocyte transplantation as an alternative is also associated with a limited cell supply and minimal engraft efficacy [2]. Another alternative therapy is therefore required urgently for hepatic fibrosis and/or cirrhosis. A concept of stem cell-based tissue engineering and regenerative medicine is expected to provide novel and promising therapeutics for refractory liver diseases [3].

Human mesenchymal stem cells (MSCs) exhibit self-renewal and multipotency into a variety of mature cells, including hepatocytes [4]. Human MSCs have been identified in a variety of human tissues, including bone marrow [5], adipose tissue [6], umbilical cord blood [7], amniotic fluid stem cells [8], and dental pulp tissue [9]. Recent studies also evaluate immunomodulatory effects of MSCs [10]. MSCs are therefore considered a feasible cell source for tissue engineering and regenerative medicine [11]. Some clinical phase I, I/II, and II trials have demonstrated that human MSC transplantation recovers hepatic function in liver cirrhosis patients [12–14], indicating that human MSCs might be a promising candidate for treatments of liver dysfunction.

Stem cells from human exfoliated deciduous teeth (SHED) are a major focus area in tissue engineering and regenerative medicine. SHED are discovered in remnant dental pulp tissues of human exfoliated deciduous teeth, and share MSC characteristics, including fibroblastic features, clonogenicity, cell surface antigen expression, cell proliferative capacity, and multidifferentiation potency [15]. SHED also modulate immune responses of interleukin-17-producing helper T (Th17) cells, regulatory T cells (Tregs), and dendritic cells [16, 17]. Recent studies have evaluated the latent potential of SHED in tissue engineering for bone regeneration [18, 19] and cell-based therapy for a variety of refractory systemic diseases, including systemic lupus erythematous, spinal cord injury, Parkinson’s disease, and diabetes [16, 20–22]. Furthermore, cryopreservation of dental pulp tissues from human deciduous teeth has succeeded [23].

Accumulating evidence has demonstrated that a variety of human MSCs, including bone marrow-derived, adipose tissue-derived, umbilical cord blood-derived, and Wharton’s jelly-derived MSCs, are capable of differentiating into hepatocyte-like cells in vivo in animal models of hepatic failure [24–26]. Advanced tissue engineering techniques accelerate a transdifferentiation ability of human MSCs into hepatocytes [27, 28]. In comparison with other human tissues, exfoliated deciduous teeth offer significant advantages of less ethical controversies and readily accessible source, easy and minimally invasive collection, and retain high stem cell potential such as cell proliferation, multipotency, and immunomodulatory functions [14–16], even after cryopreservation [23]. Recently, many investigators have investigated a SHED bank for allogenic cell therapy, as well as autologous cell therapy [23, 29, 30]. Exfoliated deciduous teeth might therefore be a feasible cell source for MSC-based therapy for both pediatric and adult patients with liver dysfunction.

Although SHED are known to be capable of differentiating into hepatocyte-like cells in vitro [31], they have not been evaluated for their in vivo hepatogenic capacity or therapeutic efficacy in liver disorders. In this study, we reveal that SHED transplantation recovers the liver dysfunction of carbon tetrachloride (CCl_4_)-treated mice. The engrafted SHED convert directly into human hepatocyte-like cells without fusion in fibrous livers of CCl_4_-treated mice. Furthermore, these in vivo SHED-converted hepatocyte-like cells participate in the hepatic recovery via both direct (tissue replacement) and indirect (anti-fibrotic and anti-inflammatory effects) integration in CCl_4_-injured mouse livers.

## Methods

### Ethics statement and human subjects

Human samples were collected as discarded biological/clinical samples from healthy pediatric donors (5–7 years old) in the Department of Pediatric Dentistry of Kyushu University Hospital, Fukuoka, Japan. Procedures using human samples were conducted in accordance with Declaration of Helsinki, and were approved by Kyushu University Institutional Review Board for Human Genome/Gene Research (Protocol Number: 393-01). Written informed consent was obtained from each parent on behalf of the child donors. All animal experiments were approved by Institutional Animal Care and Use Committee of Kyushu University (Protocol Number: A21-044-1).

### Isolation and culture of SHED

Isolation and culture of SHED were performed according to our previous reports [16, 23]. The detailed method is described in Additional file 1. To confirm whether our isolated cells were MSCs, the obtained passage 3 (P3) cells were assessed by a flow cytometric analysis as described previously [16]. The P3 cells were also cultured under osteogenic, chondrogenic, and adipogenic conditions as described previously [23]. The P3 cells were positive for CD146, CD73, CD105, and CD90, but negative for hematopoietic markers (CD34, CD45, CD14, and CD11b) (Figure S1A in Additional file 2). The P3 cells also exhibited multipotency into three types of classical mesenchymal lineage cells (Figure S1B–G in Additional file 2). These phenotypes indicated that our isolated SHED fulfilled minimal and standard criteria for MSCs [32]. The P3 cells were therefore used for further experiments in this study.

### Chronic liver fibrosis model in mice

**Fig. 1:**
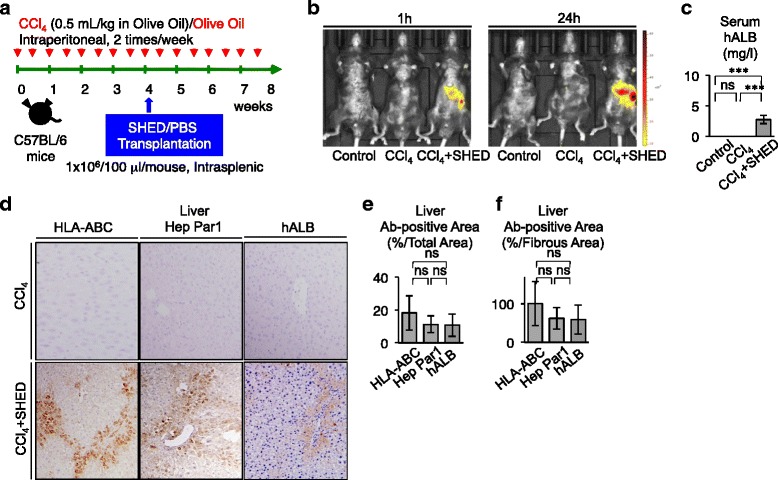
SHED differentiate into human hepatocyte-like cells in recipient livers of CCl_4_-treated mice. **a** Schema of CCl_4_ treatment and SHED transplantation in mice. C57BL/6 mice intraperitoneally received CCl_4_ (0.5 ml/kg) or olive oil only twice a week (*red arrows*). Four weeks after the treatment, SHED (1 × 10^6^) were transplanted into the CCl_4_-treated mice through the spleen. Phosphate-buffered saline (*PBS*) was infused as the control for the transplantation. **b** In vivo monitoring of transplanted DiR-labeled SHED in CCl_4_-treated mice 1 hour (1h) or 24 hours (24h) after the infusion. Dorsal position. **c** Enzyme-linked immunosorbent assay of human albumin (*hALB*) in the recipient serum. **d**–**f** Distribution of transplanted SHED in recipient livers. Immunohistochemistry with anti-human HLA-ABC, anti-hepatocyte paraffin 1 (Hep Par1), or anti-hALB antibody. Representative images. **d** Counterstaining with hematoxylin. The human HLA-ABC, hepatocyte paraffin 1, or hALB antibody positive area. Immunopositive area shown as the ratio to **e** the total area or **f** the fibrous area. **c**, **e**, **f** *n* = 5 for all groups. *P <0.05 and ***P <0.005. *ns* no significance. Graph bars show mean ± SD. Control, olive oil-injected group; CCl_4_, CCl_4_-treated group; CCl_4_ + SHED, SHED-transplanted CCl_4_-treated group. *Ab* antibody, *CCl*_*4*_ carbon tetrachloride, *HLA* human leukocyte antigen, *SHED* stem cells from human exfoliated deciduous teeth

A mixture of CCl_4_ (0.5 ml/kg body weight; Wako Pure Chemicals, Osaka, Japan) and olive oil (1:4 volume/volume; Wako Pure Chemicals) was injected intraperitoneally into C57BL/6J mice (male, 8 weeks old; Kyudo, Tosu, Japan) twice a week during this experimental period (see Fig. 1a). Age-matched and sex-matched mice injected with olive oil (Wako Pure Chemicals) were used as controls for primary (*n* = 5) and secondary (*n* = 5) transplantation.

### Primary transplantation of SHED

One million SHED (P3) suspended in 100 μl phosphate-buffered saline (PBS) were intrasplenically transplanted into mice treated with CCl_4_ for 4 weeks (*n* = 5) (Fig. 1a). As a control, 100 μl PBS were infused intrasplenically into mice treated with CCl_4_ for 4 weeks (*n* = 5). The mice continuously received CCl_4_ twice a week for an additional 4-week treatment after the transplantation. All of the animals were sacrificed to harvest the livers and peripheral blood.

### Colorimetric analysis and enzyme-linked immunosorbent assay of mouse serum and liver samples

Serum alkaline phosphatase (ALP), alanine aminotransferase (ALT), aspartate aminotransferase (AST), and total bilirubin were measured with a Multiskan GO microplate spectrophotometer (Thermo Scientific, Waltham, MA, USA) using commercially available kits according to the manufacturer’s protocol: ALP, LabAssay ALP Kit (Wako Pure Chemicals); ALT and AST, Transaminase CII-Test Kit (Wako Pure Chemicals); and total bilirubin, Bilirubin QuantiChrom Assay Kit (BioAssay Systems, Hayward, CA, USA). Liver hydroxyproline contents were measured with a Multiskan GO microplate spectrophotometer (Thermo Scientific) using a Hydroxyproline Assay Kit (Biovision, Milpitas, CA, USA). Serum mouse interleukin (IL)-6, IL-10, IL-17, transforming growth factor β1 (TGF-β1), and tumor necrosis factor alpha (TNFα) were also measured using Quantikine ELISA kits (R&D Systems, Minneapolis, MN, USA).

### Histological and immunohistochemical analyses of mouse liver tissues

Tissue preparation, Masson’s trichrome staining, and immunohistochemical staining were performed as described in Additional file 1. The sections were observed under an Axio Imager M2 (Zeiss, Oberkochen, Germany) for morphometric assays, and five representative images from each mouse were selected randomly and were used to measure a percentage of fibrous tissue area or primary antibody-positive area using ImageJ software (NIH, Bethesda, MD, USA). Trichrome stained sections were analyzed to score the amount of liver disease using Ishak scoring [33].

### Double immunofluorescence

Double immunofluorescent staining was performed as described in Additional file 1. The sections were observed under an Axio Imager M2 (Zeiss).

### Quantitative real-time RT-PCR assay

Total RNAs were extracted and treated as described in Additional file 1. Real-time RT-PCR was subsequently performed using a TaqMan Gene Expression Master Mix (Applied Biosystems, Foster City, CA, USA) and target TaqMan probes (Applied Biosystems) (Table S1 in Additional file 3) with a Light Cycler 96 (Roche, Indianapolis, IN, USA). 18S ribosomal RNA was used for normalization.

### Sorting of HLA-ABC-positive or HLA-negative cells from liver tissues of CCl_4_-treated mice transplanted with SHED

Livers of primary recipients (*n* = 5) were perfused with collagenase type H (0.1 mg/ml; Worthington Biochemicals, Lakewood, NJ, USA) in PBS and gently dispersed. Single suspended cells were stained with phycoerythrin (PE)-conjugated anti-human leukocyte antigen (HLA)-ABC (eBioscience, San Diego, CA, USA) and magnetic bead-conjugated anti-PE antibodies (Miltenyi Biotec, Bergisch Gladbach, Germany). They were magnetically sorted using a MidiMACS separator (Miltenyi Biotec) equipped with a LD column (Miltenyi Biotec), and the positive and negative fractions were collected separately.

### Cell fusion assay in HLA-positive cells

Magnetically sorted HLA-ABC-positive and HLA-negative fractions were stained with PE-conjugated anti-human major histocompatibility complex (MHC) class I HLA-ABC (eBioscience) and allophycocyanin (APC)-conjugated anti-mouse major MHC class I H-2Kb (eBioscience) antibody. The cells were measured with a FACS Verse flow cytometer (BD Biosciences, San Jose, CA, USA), and were analyzed by BD FACS Suite software (BD Biosciences).

### Human-specific genome assay in HLA-positive cells

Genomic DNA was extracted from HLA-ABC-positive and HLA-negative fractions using a DNeasy Blood and Tissue Kit (Qiagen, Venlo, the Netherlands), and was amplified with a T-100 thermal cycler (Bio-Rad, Hercules, CA, USA) using Quick Taq HS DyeMix (TOYOBO, Osaka, Japan) and specific primer pairs by PCR assay. The specific primer pairs are presented in Table S2 in Additional file 3.

### Characterization of HLA-positive cells as human hepatocytes

Sorted HLA-ABC-positive cells were cultured with Iscove’s modified Dulbecco’s medium (Invitrogen, Waltham, MA) supplemented with epidermal growth factor (EGF) (20 ng/ml; PeproTech, Rocky Hill, NJ, USA), fibroblast growth factor 2 (FGF2) (10 ng/ml; PeproTech), and hepatocyte growth factor (HGF) (20 ng/ml; PeproTech). Some cultures were stained with toluidine blue.

Expression of human hepatocyte-specific genes in HLA-positive cells was analyzed by RT-PCR with a T-100 thermal cycler (Bio-Rad) as described previously [16, 23]. The specific primer pairs are presented in Table S2 in Additional file 3. HepG2 cells (Riken, Tsukuba, Japan) were used as positive control. Human albumin and urea in the culture supernatants of HLA-positive cells were measured with a Multiskan GO microplate spectrophotometer (Thermo Scientific) using a Human Albumin ELISA Quantitation Set (AssayPro, St Charles, MO, USA) and a QuantiChrom Urea Assay Kit (Bioassay Systems), respectively.

### Secondary transplantation of HLA-ABC-positive or HLA-negative cells sorted from liver tissues of CCl_4_-treated mice with primary transplantation of SHED

To understand whether SHED-derived in vivo-converted hepatocyte-like cells express hepatic function in vivo*,* we performed a secondary transplantation of the SHED-derived in vivo*-*converted hepatocyte-like cells into CCl_4_-damaged mice. The mice (*n* = 5 each) were treated with CCl_4_ for 4 weeks, and were then transplanted with 1 million HLA-positive or HLA-negative cells via the spleen and continuously received CCl_4_ twice a week for an additional 4-week treatment after the transplantation (shown in Fig. 6a). We also used CCl_4_-treated mice and nontreated mice without the cell transplant (*n* = 5 each). Finally, the peripheral blood serum and liver samples were harvested, and used for further experiments.

### In vivo monitoring of transplanted cells

Cells were labeled with near-infrared (NIR) lipophilic carbocyanine membrane dye, 1,1-dioctadecyl-3,3,3,3-tetramethylindotricarbocyanine iodide (DiR). The cells (1 × 10^7^ in 10 ml PBS) were incubated with XenoLight DiR NIR Fluorescent Dye (10 μg/ml; Perkin Elmer, Waltham, MA, USA) for 30 minutes at 37 °C, and were then washed twice with PBS. In vivo optical imaging was performed to detect the transplanted cells. The labeled cells (1 ×10^6^ in 100 μl PBS) were infused intrasplenically into CCl_4_-pretreated mice (*n* = 5). As a control for cell transplantation, nonlabeled SHED (1 × 10^6^ in 100 μl PBS) were infused into CCl_4_-pretreated mice via the spleen (*n* = 5). Ventral images were captured from each animal group after 1 or 24 hours under an optical in vivo imaging system IVIS Lumina III (Perkin Elmer), and were analyzed using living image software (Perkin Elmer).

### Statistical analysis

Statistical results are expressed as mean ± standard deviation (SD). Multiple group comparison was analyzed by one-way repeated-measures analysis of variance followed by the Tukey post hoc test using PRISM 6software (GraphPad, Software, La Jolla, CA, USA). *P <*0.05 was considered significant.

## Results

### Transplanted donor SHED are capable of homing and differentiating into human hepatocyte-like cells in recipient livers of CCl_4_-injured mice

Mouse livers showed fibrosis after 4 weeks of treatment with CCl_4_ (data not shown). To address a therapeutic potential of SHED for liver disorders, SHED (1 × 10^6^ per mouse) were intrasplenically injected into mice that had been treated with CCl_4_ for 4 weeks (Fig. 1a). We first investigated whether transplanted SHED were capable of engrafting in the CCl_4_-treated mouse liver parenchyma. DiR-labeled SHED were infused into a spleen of CCl_4_-treated mice. In vivo imaging demonstrated that the intensity of DiR was detected on the liver, as well as the spleen, 1 hour after transplantation (Fig. 1b). The signals were enhanced in both the liver and spleen 24 hours after transplantation (Fig. 1b). Non-CCl_4_-treated mice and non-SHED-infused CCl_4_-treated mice expressed no signal at 1 and 24 hours after transplantation (Fig. 1b). By the carboxyfluorescein diacetate succinimidyl ester (CFSE)-labeled cell trace technique, CFSE-labeled SHED were detected in CCl_4_-damaged mouse liver 1 day after the transplantation (Figure S2 in Additional file 2). Our immunohistochemical analysis also detected positive immunoreactions to anti-HLA-ABC antibody in spleens of CCl_4_-damaged mice, but negative immunoreaction to anti-HLA-ABC antibody in spleens of CCl_4_-damaged mice (Figure S3B, C in Additional file 2). In addition, no immunoreaction to anti-HLA-ABC and anti-hepatocyte paraffin 1 antibodies was detected in the kidneys and lungs of CCl_4_-damaged mice (Figure S3B, C in Additional file 2). These findings suggested that DiR-labeled SHED were recruited to CCl4-damaged liver from the transplanted site, the spleen.

**Fig. 2:**
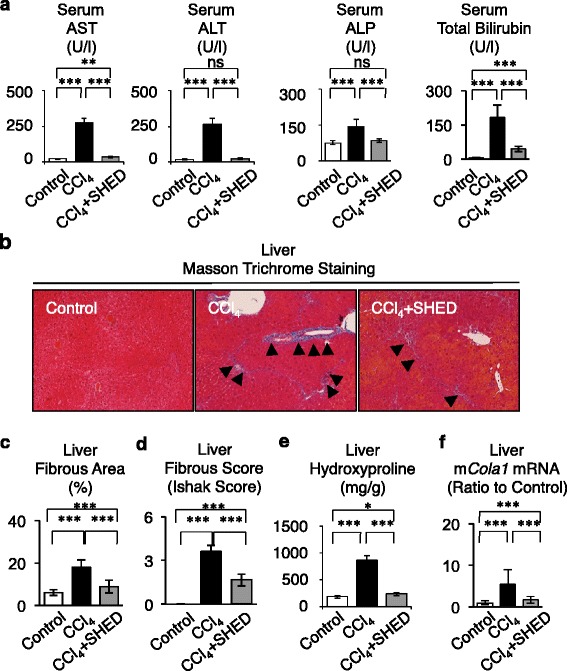
SHED ameliorate the hepatic dysfunction in recipient livers of CCl_4_-treated mice. **a** Serum assays for the hepatic function. **b**–**f** Liver fibrosis assays. **b** Representative images of livers. Masson Trichrome staining. *Arrowheads*, fibrous deposition. **c** Fibrotic area. **d** Fibrotic score. **e** Hydroxyproline assay in recipient livers. **f** Real-time RT-PCR analysis of mouse type I collagen (*mCola1*) mRNA in recipient livers. **a**, **c**–**f** *n* = 5 for all groups. Control, olive oil-injected group; CCl_4_, CCl_4_-treated group; CCl_4_ + SHED, SHED-transplanted CCl_4_-treated group. *P <0.05 and ***P <0.005. *ns* no significance. Graph bars show the mean ± SD. *ALT* alanine aminotransferase, *ALP* alkaline phosphatase, *AST* aspartate aminotransferase, *CCl*_*4*_ carbon tetrachloride, *SHED* stem cells from human exfoliated deciduous teeth

To confirm in vivo homing of transplanted SHED, peripheral blood serum and liver tissues were harvested from SHED-transplanted CCl_4_-treated mice, nontransplanted CCl_4_-treated mice, and non-CCl_4_-treated mice in week 8. Enzyme-linked immunosorbent assay (ELISA) detected human albumin in the serum of SHED-transplanted CCl_4_-treated mice, but not in both nontransplanted CCl_4_-treated mice and non-CCl_4_-treated mice (Fig. 1c). An immunohistochemical assay demonstrated that HLA-ABC-positive cells with a cuboidal shape were found in the interlobular and portal areas (Fig. 1d), which corresponded to the fibrotic region in CCl_4_-injured liver tissues (Fig. 2b). The HLA-ABC-positive cells occupied 16.27 ± 10.17 % of the recipient liver tissues (Fig. 1e). Furthermore, to verify whether the transplanted donor cells differentiated into human hepatocytes in recipient livers, immunohistochemical assay was performed using human hepatocyte-specific hepatocyte paraffin 1 and human albumin-specific antibodies. The hepatocyte paraffin 1-positive and human albumin-positive cells were distributed in interlobular and portal areas of the recipient livers similar to the HLA-ABC-positive cells (Fig. 1d), and were expressed in 11.39 ± 4.58 % and 10.73 ± 6.18 % of the recipient liver tissues, respectively (Fig. 1e). The hepatocyte paraffin 1-positive and human albumin-positive areas tended to be less than the HLA-ABC-positive area, but not significant (Fig. 1e). No immunoreactivity against HLA-ABC, hepatocyte paraffin 1, or human albumin was found in the liver tissue of nontransplanted CCl_4_-induced mice (Fig. 1d) or in control mice (data not shown). No immunoreactivity against HLA-ABC, hepatocyte paraffin 1, or human albumin is found for liver sections treated with nonimmune IgG instead of the primary antibodies (Figure S4 in Additional file 2). Positive immunoreaction to anti-HLA-ABC, anti-hepatocyte paraffin 1, and anti-human albumin antibodies was detected in almost of parenchymal cells of human liver tissues (Figure S5 in Additional file 2), but human liver tissues expressed negative immunoreaction to nonimmune mouse IgG (Figure S5 in Additional file 2). These results indicated that donor SHED showed an in vivo capacity of engrafting and differentiating into human hepatocyte-like cells in the recipient livers of CCl_4_-injured mice.

### SHED transplantation decreased CCl_4_-induced chronic fibrosis in mouse livers

To address whether SHED have therapeutic potential for liver disorders, SHED-transplanted CCl_4_-treated mice, as well as nontransplanted (PBS-injected) CCl_4_-treated mice, received continuous CCl_4_ injections for an additional 4 weeks (Fig. 1a). In week 8, the nontransplanted mice showed severe fibrous liver dysfunction (Fig. 2). A biochemical serum assay revealed that SHED transplantation markedly recovered the damaged liver functions (Fig. 2a). Masson trichrome staining showed that SHED transplantation reduced CCl_4_-enhanced fibrous deposition in the liver (Fig. 2b, c). The fibrous tissue area occupied 5.98 ± 1.35 %, 18.16 ± 3.36 %, and 8.89 ± 3.07 % of the recipient liver tissues in control mice, nontransplanted CCl_4_-treated mice, and SHED-transplanted CCl_4_-treated mice, respectively (Fig. 2c). The degree of hepatic fibrosis by Ishak score [31] was 0 ± 0, 3.60 ± 0.43, and 1.67 ± 0.41 of the recipient liver tissues in control mice, nontransplanted CCl_4_-treated mice, and SHED-transplanted CCl_4_-treated mice, respectively (Fig. 2d). Colorimetric and real-time PCR assays revealed that SHED transplantation significantly reduced the hydroxyproline content and collagen production in the CCl_4_-damaged liver tissues (Fig. 2e, f). Interestingly, HLA-ABC, hepatocyte paraffin 1, or human albumin-positive cells captured a similar area to the fibrous deposit region in the liver of nontransplanted CCl_4_-treated mice (Fig. 1d–f). To confirm the in vivo hepatogenic differentiation capacity and therapeutic efficacy of SHED in recipient CCl_4_-injured livers, we infused pediatric human gingival fibroblasts as a control for SHED transplantation in CCl_4_-treated mice (Figure S6A in Additional file 2). Immunohistochemical assay showed that no HLA-ABC, hepatocyte paraffin 1, or human albumin-positive human cells were detected in the recipient CCl_4_-damaged liver tissues (Figure S6B in Additional file 2). Biochemical assays demonstrated that human gingival fibroblast infusion did not recover the impaired hepatic function in CCl_4_-injected mice (Figure S6C in Additional file 2). Taken together, these findings indicated that SHED transplantation suppressed CCl_4_-enhanced fibrous deposition in the liver of CCl_4_-treated mice, and suggested that SHED directly/spontaneously transdifferentiated into human hepatocytes in CCl_4_-damaged livers.

**Fig. 3:**
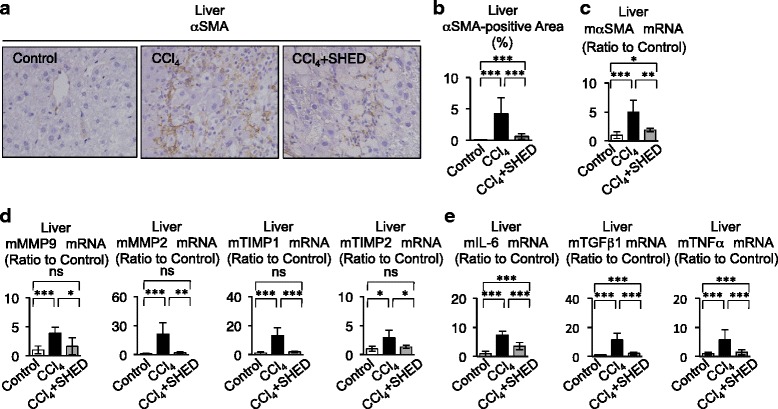
SHED transplantation suppresses the activation of hepatic stellate cells in livers of CCl_4_-treated mice. **a**–**c** Expression of alpha smooth muscle actin (*αSMA*). **a** Immunohistochemical staining with anti-αSMA antibody in recipient livers. **b** αSMA-positive area. **c** Real-time RT-PCR analysis of mouse αSMA (*mαSMA*) mRNA. **d**, **e** Real-time RT-PCR analysis**. d** Expression of mouse MMP9 and MMP2 (*mMMP9*, *mMMP2*) and mouse TIMP1 and TIMP2 (*mTIMP1*, *mTIMP2*) mRNA in recipient livers. **e** Expression of mouse interleukin-6 (*mIL-6*), mouse transforming growth factor β1 (*mTGFβ1*), and mouse tumor necrosis factor alpha(*mTNFα*) mRNA in recipient livers. **b**–**e** *n* = 5 for all groups. *P <0.05, **P <0.01, and ***P <0.005. *ns* no significance. Graph bars show the mean ± SD. Control, olive oil-injected group; CCl_4_, CCl_4_-treated group; CCl_4_ + SHED: SHED-transplanted CCl_4_-treated group. *CCl*_*4*_ carbon tetrachloride, *MMP* matrix metalloproteinase, *SHED* stem cells from human exfoliated deciduous teeth, *TIMP* tissue inhibitor of matrix metalloproteinase

Activation of hepatic stellate cells is a crucial event required to initiate and promote hepatic fibrosis, followed by producing and remodeling of type I collagen by matrix metalloproteinases (MMPs) and tissue inhibitors of metalloproteinase (TIMPs) [34]. We therefore examined the kinetics of activated hepatic stellate cells after SHED transplantation in recipient livers 8 weeks after the first CCl_4_ injection. Immunohistochemical analysis indicated that SHED transplantation decreased the area of alpha smooth muscle actin (αSMA)-positive cells, which indicated activated hepatic stellate cells, in the CCl_4_-injured liver tissues (Fig. 3a, b). A real-time PCR assay also demonstrated that SHED transplantation significantly reduced the expression of αSMA mRNA (Fig. 3c) and markedly suppressed CCl_4_-induced MMP2, MMP9, TIMP1, and TIMP2 mRNA expression (Fig. 3d) in the injured livers.

**Fig. 4:**
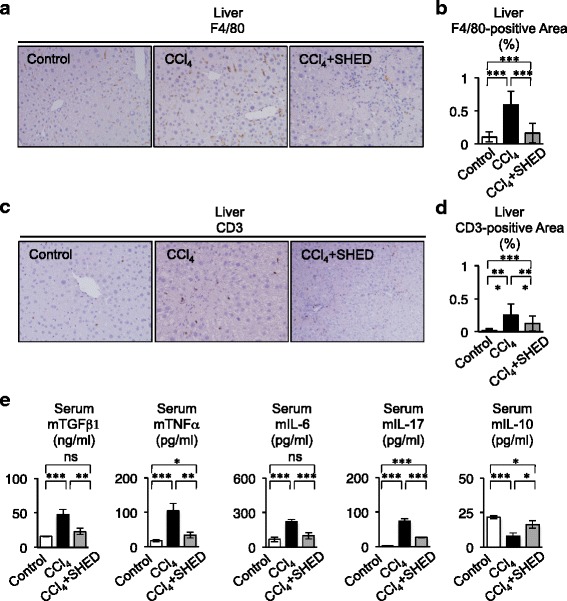
SHED transplantation inhibits the enhanced distribution of Kupffer cells and T cells in recipient livers of CCl_4_-treated mice. **a**–**c** Expression of F4/80 and CD3 in recipient livers. Immunohistochemical staining with anti-F4/80 (**a**) and anti-CD3 antibodies (**c**). The F4/80-positive (**b**) and CD3-positive (**d**) area. **e** ELISA of recipient serum. **b**, **d**, **e** *n* = 5 for all groups. *P <0.05, **P <0.01, and ***P <0.005. Graph bars show the means ± SD. Control, olive oil-injected control group; CCl_4_, CCl_4_-treated group; CCl_4_ + SHED, SHED-transplanted CCl_4_-treated group. *CCl*_*4*_ carbon tetrachloride, *mIL* mouse interleukin, *mTGFβ1* mouse transforming growth factor β1, *mTNFα* mouse tumor necrosis factor alpha, *SHED* stem cells from human exfoliated deciduous teeth

Kupffer cells and T lymphocytes and the fibrotic and inflammatory cytokines, such as TGF-β, TNFα, IL-6, and IL-17, produced by them are also involved in the progression of hepatic fibrosis and activation of hepatic stellate cells [34, 35]. By immunohistochemical assays, CCl_4_ treatment markedly induced infiltration of F4/80-positive and CD3-positive cells in the liver, which indicate Kupffer cells and/or macrophages and T lymphocytes, respectively, compared with non-CCl_4_-treated livers (Fig. 4a–c). SHED transplantation suppressed the altered distribution of F4/80-positive and CD3-positive cells in the CCl_4_-treated livers (Fig. 4a–d). Further histochemical analysis demonstrated that SHED transplantation did not induce any heavy infiltration of lymphocyte-like cells, and did not cause any severe change of structural components in other tissues such as the kidneys, lungs, and spleens of CCl_4_-treated mice with SHED (Figure S3A in Additional file 2). Real-time PCR and ELISA studies demonstrated that SHED transplantation reduced the expression of TGF-β1, TNFα, and IL-6 mRNAs in the CCl_4_-induced fibrous livers (Fig. 3e), and suppressed the elevation of IL-6, TGF-β, and TNFα in the serum of CCl_4_-treated mice (Fig. 4e). SHED transplantation reduced the proinflammatory IL-17 expression and recovered the decreased anti-inflammatory IL-10 expression in the CCl_4_-treated livers (Fig. 4e). Taken together, these findings indicated that transplanted SHED might exhibit anti-fibrotic and anti-inflammatory effects against liver fibrosis by suppressing the activation of hepatic stellate cells, Kupffer cells/macrophages, and T cells.

### Donor SHED are capable of differentiating into human hepatocyte-like cells without fusion in CCl_4_-injured mouse livers

**Fig. 5:**
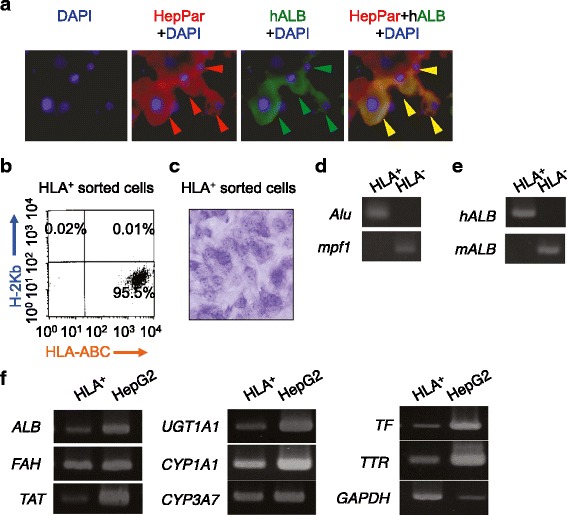
SHED-derived HLA-ABC-positive cells purified from primary recipient livers of CCl_4_-treated mice express hepatocyte-specific genes without host-cell fusion*.* **a** Double-immunofluorescent staining patterns for HepPar and human albumin (*hALB*) in CCl_4_-injured liver tissues transplanted with SHED. **b** Flow cytometric analysis of magnetically sorted HLA-ABC-positive (*HLA* ^+^) cells stained with PE-conjugated anti-human HLA-ABC and APC-conjugated anti-mouse H-K2^b^ antibodies. **c** Morphology of sorted HLA^+^ cells. Toluidine blue staining. **d** Genomic DNA assay. **e** RT-PCR analysis of hALB and mouse albumin (*mALB*) mRNAs. **f** RT-PCR analysis of human hepatocyte-specific genes. *ALB* albumin, *Alu* human-specific Alu gene, *CCl*_*4*_ carbon tetrachloride, *CYP1A1* cytochrome P450 1A1, *CYP3A7* cytochrome P450 3A7, *DAPI* 4′,6-diamidino-2-phenylindole, *FAH* fumarylacetoacetate hydrolase, *GAPDH* human glyceraldehyde 3-phosphate dehydrogenase, *HepG2* human hepatoma cell line, *HepPar1* human hepatocyte specific HepParaffin 1 antigen, *HLA* human leukocyte antigen, *HLA* ^–^ HLA-ABC-negative cells, *mpf1* mouse-specific Pf1 gene, *SHED* stem cells from human exfoliated deciduous teeth, *TAT* tyrosine aminotransferase, *TF* transferrin, *TTR* transthyretin, *UGT1A1* uridine 5′-diphospho-glucuronosyltransferase 1A1

Transplanted bone marrow cells fuse with host hepatocytes in damaged livers [36, 37], but bone marrow MSCs differentiate into hepatocytes without cell fusion in recipients [24]. Using dual immunofluorescent staining using human specific antibodies to hepatocyte paraffin 1 and albumin, we demonstrated that double positive cells to hepatocyte paraffin 1 and human albumin were found in liver tissues of CCl_4_-injured mice with SHED transplantation (Fig. 5a). However, it was unclear whether the double positive cells were fused with host cells or not; a possibility of cell fusion between donor SHED and recipient hepatocytes remained. To evaluate whether the in vivo converted SHED-derived human hepatocyte-like cells were fused with host hepatocytes, we isolated human cells from recipient livers of SHED-transplanted CCl_4_-treated mice (Figure S7 in Additional file 2). Pan-liver cells were isolated from the recipient livers with the collagenase digestion method, and stained with anti-HLA-ABC antibody. The HLA-ABC-positive cells were magnetically sorted to collect separately from HLA-ABC-negative cells. Flow cytometric analysis confirmed that the HLA-ABC-positive fraction was 95.5 ± 4.43 % positive to HLA-ABC, but negative to mouse H-2Kb (Fig. 5b). Double positive cells were also not detected (Fig. 5b). On the other hand, the HLA-ABC-negative fraction was 96.3 ± 5.68 % positive to H-2Kb, but 0 % to HLA-ABC (data not shown). The HLA-ABC-positive cells maintained under EGF, FGF2, and HGF stimulation for 3 days showed a cuboidal shape on the dishes by toluidine blue staining (Fig, 5c). A genomic DNA assay demonstrated that a human specific gene, *Alu*, was detected only in HLA-ABC-positive cells, but not in HLA-ABC-negative cells (Fig. 5d). On the other hand, a mouse specific gene, *mpf1*, was not detected in HLA-ABC-positive cells, but was found in HLA-ABC-negative cells (Fig. 5d). RT-PCR analysis also demonstrated that human albumin gene was detected only in HLA-ABC-positive cells, but not in HLA-ABC-negative cells, while mouse albumin gene was expressed in HLA-ABC-negative cells, but not in HLA-ABC-positive cells (Fig. 5e). These data indicate that transplanted SHED were directly transdifferentiated into human hepatocytes without fusion with recipient mouse hepatocytes.

Further RT-PCR assay demonstrated that the purified HLA-ABC-positive cells expressed human hepatocyte-specific genes, albumin, cytochrome P450 1A1, cytochrome P450 3A7, fumarylacetoacetase, tyrosine aminotransferase, uridine 5′-diphospho (UDP)-glucuronosyltransferase, transferrin, and transthyretin (Fig. 5f). However, the expression levels of human hepatocyte-specific genes in the purified HLA-ABC-positive cells were lower when compared with human hepatocyte cell line HepG2 (Fig. 5f). By ELISA and colorimetric assay, human albumin, urea, and blood urea nitrogen were detected at 4.8 ± 0.085 ng/ml, 0.47 ± 0.01 mg/dl, and 0.22 ± 0.005 mg/dl, respectively, in the culture supernatant of HLA-positive cells cultured with EGF, FGF2, and HGF stimulation for 3 days. Taken together, these findings indicate that SHED might show a potential for transdifferentiating into functional human hepatocytes, at least partially, without fusing with host mouse hepatocytes in fibrotic livers of CCl_4_-treated mice.

### Secondary transplantation of SHED-derived human hepatocyte-like cells purified from primary CCl_4_-injured recipient livers recovered hepatic dysfunction of CCl_4_-treated mice

**Fig. 6:**
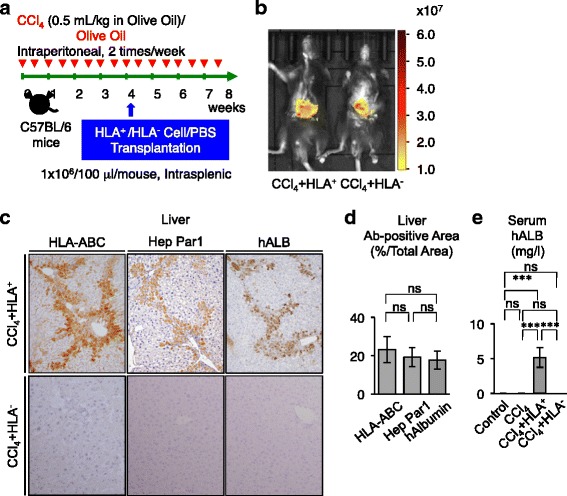
Secondary transplanted primary HLA-ABC-positive cells home to CCl_4_-treated recipient livers*.* **a** Schema of secondary transplantation of primary HLA-ABC^+^/HLA-ABC^–^ cells into C57BL/6 mice. HLA-ABC^+^/HLA-ABC^–^ cells (1 × 10^6^) or phosphate-buffered saline (*PBS*) were infused into the mice that had intraperitoneally received CCl_4_ (0.5 ml/kg) or olive oil only twice a week (*red arrows*). **b** In vivo monitoring of DiR-labeled HLA-ABC-positive (*HLA* ^+^) and HLA-negative (*HLA* ^–^) cells in CCl_4_-treated mice 24 hours (24h) after the infusion. **c**, **d** Distribution of transplanted HLA^+^ and HLA^–^ cells in the secondary recipient livers. Immunohistochemistry with anti-human HLA-ABC (HLA-ABC), anti-hepatocyte paraffin 1 (Hep Par1), or anti-human albumin (hALB) antibody. Representative images. **c** Counterstaining with hematoxylin. The human HLA-ABC, hepatocyte paraffin 1, or human albumin-positive area. **d** Immunopositive area shown as the ratio to the total area. **e** ELISA of human albumin (*hALB*) in the recipient serum. **c**–**e** *n* = 5 for all groups. ***P <0.005. *ns* no significance. Graph bars show the mean ± SD. Control, olive oil-injected group; CCl_4_, CCl_4_-treated group; CCl_4_ + HLA^+^, HLA^+^ cell-transplanted CCl_4_-treated group; CCl_4_ + HLA^–^, HLA^–^ cell-transplanted CCl_4_-treated group. *Ab* antibody, *CCl*_*4*_ carbon tetrachloride, *HepPar1* human hepatocyte specific HepParaffin 1 antigen, *HLA* human leukocyte antigen

Next we examined the homing capability of SHED-derived in vivo*-*converted hepatocyte-like cells. Mice that had been treated with CCl_4_ for 4 weeks underwent secondary transplantation of purified HLA-ABC-positive cells (1 × 10^6^), as well as HLA-ABC-negative cells (1 × 10^6^), into the spleen (Fig. 6a). In vivo imaging analysis showed that strong intensity of DiR-labeled HLA-ABC-positive and DiR-labeled HLA-ABC-negative cells was observed in the livers of CCl_4_-treated mice 24 hours post transplantation (Fig. 6b). Further immunohistochemical analysis and ELISA was performed in the liver tissues and peripheral blood serum of CCl_4_-treated mice that underwent secondary transplantation with HLA-ABC-positive and HLA-ABC-negative cells, as well as of nontransplanted CCl_4_-treated mice and nonCCl_4_-treated mice, in week 8.

An immunohistochemical examination demonstrated that HLA-ABC-positive, hepatocyte paraffin 1-positive, and human albumin-positive cells were observed in the interlobular and portal regions corresponding to the fibular deposited area in liver tissues of CCl_4_-treated mice that underwent secondary transplant with HLA-ABC-positive cells 4 weeks after the primary transplant (Fig. 6c). The HLA-ABC-positive, hepatocyte paraffin 1-positive, and human albumin-positive cell areas were 23.22 ± 6.81 %, 19.31 ± 5.06 %, and 17.80 ± 4.71 % in the secondary recipient livers (Fig. 6d). The immunohistochemically positive areas expressed a similar rate to the liver fibrous area of nontransplanted CCl_4_-injured mice (Figure S8 in Additional file 2). No immunoreactivity against HLA-ABC, hepatocyte paraffin 1, or human albumin was detected in the liver tissues of CCl_4_-induced mice that underwent secondary transplant with HLA-ABC-negative cells (Fig. 6c) or in nontransplanted CCl_4_-induced mice and non-CCl_4_-induced mice (data not shown). ELISA also showed that serum human albumin was detected in CCl_4_-treated mice that underwent secondary transplant with HLA-ABC-positive cells, but not in CCl_4_-treated mice that underwent secondary transplant with HLA-ABC-negative cells, nontransplanted CCl_4_-treated mice, and non-CCl_4_-treated mice (Fig. 6e).

**Fig. 7:**
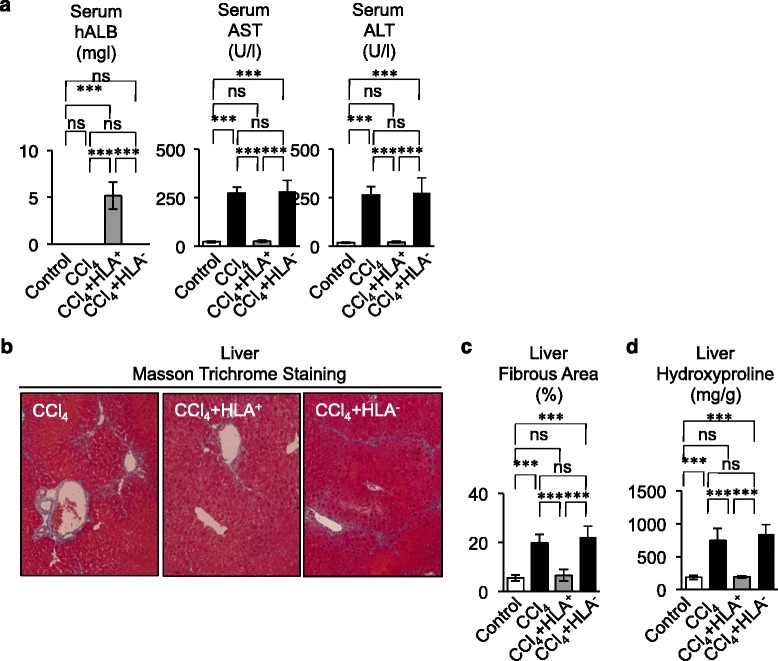
Secondary transplantation of primary HLA-ABC-positive cells ameliorates the liver dysfunction in CCl_4_-treated recipient livers*.* **a** Serum assays for the hepatic function. **b**–**d** Liver fibrosis assays. **b** Representative images of livers. Masson Trichrome staining. **c** Fibrotic area. **d** Hydroxyproline assay in recipient livers. **a, c, d** *n* = 5 for all groups. ***P <0.005. *ns* no significance. Graph bars show the mean ± SD. Control, olive oil-injected group; CCl_4_, CCl_4_-treated group; CCl_4_ + HLA^+^, HLA^+^ cell-transplanted CCl_4_-treated group; CCl_4_ + HLA^–^, HLA^–^ cell-transplanted CCl_4_-treated group. *AST* aspartate aminotransferase, *ALT* alanine aminotransferase, *CCl*_*4*_ carbon tetrachloride, *hALB* human albumin, *HLA* human leukocyte antigen

To evaluate a therapeutic efficacy of SHED-derived in vivo*-*converted hepatocyte-like cells, peripheral blood serum and liver tissues were harvested from the mice in week 8. Serum assay demonstrated that the secondary transplantation of primary HLA-ABC-positive cells recovered hepatic markers of CCl_4_-treated mice (Fig. 7a; Figure S9 in Additional file 2). Masson trichrome staining and hydroxyproline content assay demonstrated that the secondary transplantation of primary HLA-ABC-positive cells reduced the production and deposition of fibrous matrix (Fig. 7b–d; Figure S10A in Additional file 2). By real-time RT-PCR, expression of mouse type I collagen mRNA was also suppressed in the secondary recipient liver transplanted with HLA-ABC-positive cells compared with the nontransplanted recipient livers (Figure S10B in Additional file 2). On the other hand, the secondary transplantation of HLA-ABC-negative cells did not restore the hepatic function and fibrous tissue deposition in CCl_4_-injured livers (Fig. 7; Figures S9 and S10 in Additional file 2).

**Fig. 8:**
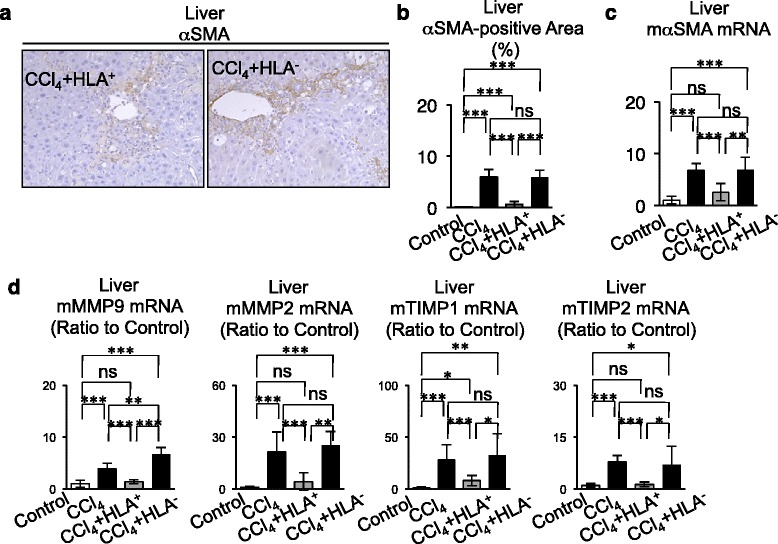
Secondary transplantation of primary HLA-ABC-positive cells suppresses the activation of hepatic stellate cells and induction of Kupffer cells in livers CCl_4_-treated mice. **a**–**c** Expression of alpha smooth muscle actin (*αSMA*). **a** Immunohistochemical staining with anti-αSMA antibody in recipient livers. **b** αSMA-positive area. **c** Real-time RT-PCR analysis of mouse αSMA (*mαSMA*) mRNA. **d** Real-time RT-PCR analysis of mouse MMP9 and MMP2 (*mMMP9*, *mMMP2*) and mouse TIMP1 and TIMP2 (*mTIMP1*, *mTIMP2*) mRNA in recipient livers. **b**–**d** *n* = 5 for all groups. *P <0.05, **P <0.01, and ***P <0.005. *ns* no significance. Graph bars show the mean ± SD. Control, olive oil-injected group; CCl_4_, CCl_4_-treated group; CCl_4_ + HLA^+^, HLA^+^ cell-transplanted CCl_4_-treated group; CCl_4_ + HLA^–^, HLA^–^ cell-transplanted CCl_4_-treated group. *CCl*_*4*_ carbon tetrachloride, *HLA* human leukocyte antigen, *MMP* matrix metalloproteinase, *TIMP* tissue inhibitor of matrix metalloproteinase

Moreover, by immunohistochemical and real-time PCR analyses, we demonstrated that secondary transplantation of HLA-ABC-positive cells significantly reduced the increased αSMA expression in CCl_4_-injured liver tissues (Fig. 8a–c). Further real-time PCR assay demonstrated that the secondary transplantation of HLA-ABC-positive cells markedly inhibited the enhanced MMP2, MMP9, TIMP1, and TIMP2 mRNA expressions (Fig. 8d) in CCl_4_-injured livers. On the other hand, the increased distribution of αSMA-positive cells and enhanced expression of αSMA, MMP2, MMP9, TIMP1, TIMP2, TGF-β1, TNFα, and IL-6 mRNAs were not recovered in CCl_4_-treated mice that underwent secondary transplant with HLA-ABC-negative cells (Fig. 8). Taken together, these findings suggested that in vivo*-*generated hepatocyte-like cells in CCl_4_-injured livers with SHED transplantation worked functionally, at least partially, as human hepatocytes to display therapeutic efficacy for CCl_4_-induced liver fibrosis [38].

## Discussion

Severe shortage of donor organs is a major challenge for liver transplantation [1]. Because of their unique capacities for homing and hepatic differentiation, MSCs and hematopoietic stem cells have been receiving attention as a source for cell therapy as an alternative to liver transplantation [39]. Transplantation of isolated mature hepatocytes has been used as an experimental therapy for liver disease in a limited number of cases. Recently, 100 cases of hepatocyte transplantation have been reported. Clinically, hepatocyte transplants express a proven efficiency, particularly in cases of metabolic liver disease where reversal or amelioration of the characteristic symptoms of the disease is easily quantified. However, no patients are completely corrected of a metabolic liver disease for a significant amount of time by hepatocyte transplantation alone [40]. MSC transplantation [12–14], as well as hematopoietic stem cell transplantation [41, 42], can successfully treat liver failure in animal models. MSCs exhibit a greater therapeutic efficacy with regard to homing and reducing fibrosis in comparison with hematopoietic stem cells in injured livers [43, 44]. In the present study, we demonstrated that SHED transplantation improved CCl_4_-induced liver fibrosis and hepatic dysfunction via inertness of activated hepatic stellate cells and by replacement of damaged tissue with transplanted SHED-derived hepatocyte-like cells. These findings therefore suggest that SHED might be a promising MSC source for liver regeneration.

The present study demonstrated that SHED transplantation markedly suppressed not only the pathological activation of hepatic stellate cells, but also the excessive infiltration of Kupffer cells and T cells in CCl_4_-damaged mouse livers. Furthermore, SHED transplantation significantly reduced the enhanced production of fibrogenic and inflammatory factors, such as TGF-β1, TNFα, MMP2, MMP9, TIMP1, TIMP2, IL-6, and IL-17, and enhanced the expression of the anti-inflammatory factor IL-10 in CCl_4_-induced fibrous livers. Activated hepatic stellate cells contribute to liver fibrosis via abnormal production of MMP2, TIMP1, and TIMP2 through the secretion of various inflammatory cytokines from Kupffer cells and T cells [34, 35]. SHED can induce Tregs and suppress Th17 cells and monocytes/dendritic cells [16, 17]. Transplanted SHED might therefore suppress immune responses and promote anti-fibrotic regulation by affecting hepatic stellate cells, Kupffer cells, and T cells in CCl_4_-damaged mouse livers.

We speculate that a considerable number of transplanted SHED might be rejected immunologically owing to the present xenogeneic transplantation system and nonimmunosuppressive status in immunocompetent mice. We also consider a possibility that donor SHED and the differentiated hepatocytes, as well as recipient hepatocytes, might be damaged by chronic CCl_4_ stimuli. On the contrary, a result that donor SHED survived to differentiate into human hepatocytes in CCl_4_-injured liver tissues suggests that the donor cells maintained higher toxic resistance compared with recipient cells, and supports that donor SHED, at least partially, showed a tolerance to host immune response, even under nonimmunosuppressive condition, in immunocompetent mice. Furthermore, SHED transplantation did not induce any heavy infiltration of lymphocyte-like cells, as well as any change of structural components, in other tissues including the kidney, lung, and spleen of CCl_4_-treated mice. On the other hand, SHED transplantation suppressed the immune reaction in CCl_4_-treated mice. These findings support that donor SHED did not cause any graft versus host disease-like reaction. Taken together, these findings suppose that SHED might exhibit safe immunology in the present xenogeneic transplantation system. Less HLA-DR expression and active immunomodulatory function of SHED may support a low immunogenicity and can acquire immune tolerance in vivo [16, 45]. Further study will be necessary to confirm the immunological safety of SHED as a donor for allogenic transplantation, as well as autologous transplantation, for liver patients.

The liver is a site of hematopoiesis in the fetus, so bone marrow hematopoietic stem cells have been considered an origin for hepatocytes in adults [46, 47]. Transplanted hematopoietic stem cells fuse with host hepatic cells to repopulate the liver as functional hepatocytes [36, 37]. On the other hand, a nonfusion origin of human hepatocytes was proposed in mouse liver transplanted with human hematopoietic cells [48–50]. Engrafted bone marrow MSCs directly transdifferentiated into hepatocytes without cell fusion in rat livers [24]. Therefore, whether donor human cells fuse with recipient hepatic cells in mouse liver has not yet been fully understood. The presented three different approaches with a cell sorting technique of MHC class I antigen HLA-ABC-expressed human cells from the recipient mouse liver were carried out to evaluate the possibility of fusion between donor human MSCs and recipient murine hepatocytes. By flow cytometric analysis using human and mouse specific antibodies against MHC class I antigen, cell fusion of the donor cells and recipient cells was excluded. PCR analysis using human and mouse specific primers also omits the possibility of cell fusion. In a further secondary transplant assay, HLA-ABC-negative cells have in vivo differentiation capacity into human hepatocytes. These results indicate that donor-derived human hepatocytes have only human genetic and immunological properties, suggesting that cell fusion of donor SHED and recipient hepatocytes in the hepatogenic process may be a rare or nonexistent phenomenon in recipient CCl_4_-injured mice. From another point of view, cell fusion between recipient hepatocytes and hematopoietic stem cells might lead to genetic instability and formation of cancer stem cells [51]. Human MSCs exhibit a low tumorigenic potential in vivo [52] and in vitro [53]. The present findings indicate that SHED may provide an attractive and safe source for stem cell-based liver regeneration. However, a long-term in vivo experiment will be necessary to assess the safety and tumorigenic risk(s) after SHED transplantation in damaged livers.

The present immunohistochemical findings suggest that intrasplenically infused donor SHED are transported into recipient liver through the portal vein system via the splenic vein, and penetrated into CCl_4_-damaged fibrous area via the interlobular portal veins. However, the mechanism underlying in vivo homing and hepatic potential of transplanted MSCs, including SHED, remains unclear. In vivo homing and hepatic potential of MSCs might be regulated by a microenvironment of injured liver tissues. Liver contributes to a niche for hematopoietic stem cells in the fetus [54] and in patients with osteomyelofibrosis [55]. Hepatic stellate cells support hematopoiesis in fetal livers [56], and activated hepatic stellate cells release a factor associated with stem cell homing and migration, C-X-C motif chemokine 12 [57], and a factor promoting hepatocyte proliferation and differentiation, HGF [58]. In addition, hepatic stellate cells modulate a hepatogenic potential of bone marrow MSCs [59]. These previous studies suggest that activated hepatic stellate cells might function as a niche to modulate the homing and hepatic differentiation of transplanted MSCs. Further studies will be necessary to elucidate cellular and molecular mechanism(s) responsible for in vivo homing and hepatic potential of transplanted MSCs, including SHED.

In this study, purified HLA-ABC-positive cells from liver tissue of SHED-transplanted CCl_4_-treated mice confirmed the expression of several characteristics as human hepatocyte-like cells. The present secondary transplantation into CCl_4_-treated mice analysis demonstrates that purified HLA-ABC-positive cells express a homing capacity and a treatment efficacy in CCl_4_-injured mice, suggesting that in vivo-converted SHED-derived hepatocytes may function as human hepatocytes. Chimeric human livers with more than 90 % human hepatocytes are successfully developed in murine models [60, 61]. A recently reported novel tissue engineering approach generated a transplantable recellularized liver graft with human hepatocytes and MSCs using xenogeneic decellularized livers [62, 63]. The present in vivo serial transplantation assay demonstrated that SHED-derived direct-converted hepatocytes exhibit chimerism and therapeutic effect in CCl_4_-damaged mouse livers. These results suggest that in vivo-generated human hepatocyte-like cells derived from donor SHED may provide an alternative source for banking of human hepatocytes and development of human chimeric livers in vivo *and* ex vivo.

## Conclusion

In summary, this report provides a foundation for SHED-based liver regenerative medicine. Further studies will be required to elucidate whether this practical and unique approach can be applied clinically for patients with liver disorders, such as liver fibrosis, metabolic diseases, or some coagulopathies.

## Additional files

Additional file 1:
**Presents supplementary methods.** (DOC 91 kb) Additional file 2:
**Figure S1.** Showing characterization of SHED, **Figure S2** showing CFSE-labeled cell tracing of donor SHED 1 day after transplantation, **Figure S3** showing histological and immunohistochemical analyses of the kidney, lung, and spleen of CCl_4_-treated mice with splenic SHED transplantation, **Figure S4** showing immunohistochemical negative control analysis, **Figure S5** showing immunohistochemical analysis of human liver, **Figure S6** showing transplantation of human gingival fibroblasts into CCl_4_-treated mice, **Figure S7** showing the schema for cell sorting of human cells from recipient liver tissues of CCl_4_-treated mice transplanted with SHED, **Figure S8** showing human HLA-ABC, hepatocyte paraffin1 (Hep Par1), or human albumin (hALB) antibody-positive area in the recipient liver tissues of secondary transplant CCL-treated mice, **Figure S9** showing serum assays for the hepatic function in secondary transplant CCl4-treated mice, and **Figure S10** showing fibrous assay in recipient livers of secondary transplant CCl4-treated mice. (PDF 1386 kb) Additional file 3:
**Table S1.** Presenting the TaqMan primers and probes used for mouse genes used in real-time PCR, and **Table S2** presenting the primer pairs used for human and mouse genes for genomic PCR and RT-PCR. (DOC 65 kb)

## Abbreviations

ALP: Alkaline phosphatase
ALT: Alanine aminotransferase
APC: Allophycocyanin
AST: Aspartate aminotransferase
CCl_4_: Carbon tetrachloride
CFSE: Carboxyfluorescein diacetate succinimidyl ester
DiR: 1,1-Dioctadecyl-3,3,3,3-tetramethylindotricarbocyanine iodide
EGF: Epidermal growth factor
ELISA: Enzyme-linked immunosorbent assay
FGF2: Fibroblast growth factor 2
GAPDH: Glyceraldehyde 3-phosphate dehydrogenase
HGF: Hepatocyte growth factor
HLA: Human leukocyte antigen
IL: Interleukin
MHC: Major histocompatibility complex
MMP: Matrix metalloproteinase
MSC: Mesenchymal stem cell
NIR: Near infrared
P3: Passage 3
PBS: Phosphate-buffered saline
PE: Phycoerythrin
SD: Standard deviation
SHED: Stem cells from human exfoliated deciduous teeth
TGF-β1: Transforming growth factor β1
Th17: Interleukin-17-producing helper T
TIMP: Tissue inhibitor of metalloproteinase
TNFα: Tumor necrosis factor alpha
Tregs: Regulatory T cells
UDP: Uridine 5′-diphosphate
UDP: Uridine 5′-diphosphate
αSMA: Alpha smooth muscle actin
