# Supplementary material for: In vivo hepatogenic capacity and therapeutic potential of stem cells from human exfoliated deciduous teeth in liver fibrosis in mice
Source: Stem Cell Res Ther. 2015 Sep 10;6(1):171. doi: 10.1186/s13287-015-0154-6 (PMC4566368; doi:10.1186/s13287-015-0154-6)
Supplement: Additional file 1: — Presents supplementary methods. (DOC 91 kb) [file 13287_2015_154_MOESM1_ESM.doc]

**Additional file 1: Supplementary Methods**

**In vivo direct hepatogenic capacity therapeutic potential of stem cells from human exfoliated deciduous teeth in liver fibrosis in mice**

Takayoshi Yamaza^1†^, Fatima Safira Alatas^4†^, Haruyoshi Yamaza^2^, Ratih Yuniartha^4^, Junko K. Fujiyoshi^5^, Yusuke Yanagi^4^, Koichiro Yoshimaru^4^, Makoto Hayashida^4^, Toshiharu Matsuura^4^, Reona Aijima^1^, Soichiro Sonoda^1^, Fusanori Nishimura^3^, Kenji Ihara^6^, Shouichi Ohga^7^, Kazuaki Nonaka^2^, Tomoaki Taguchi^4*^

Departments of ^1^Molecular Cell Biology and Oral Anatomy, ^2^Pediatric Dentistry, and ^3^Periodontology, Kyushu University Graduate School of Dental Science, Fukuoka, Japan.

Departments of ^4^Pediatric Surgery and ^5^Pediatrics, Kyushu University Graduate School of Medical Sciences, Fukuoka, Japan.

^6^Department of Pediatrics, Faculty of Medicine, Oita University, Yuhuin, Japan.

^7^Department of Pediatrics, Faculty of Medicine and Health Sciences, Yamaguchi University, Ube, Japan.

^†^These authors contributed equally to this work.

****Correspending to:***

Tomoaki Taguchi, MD, PhD., FACS

Department of Pediatric Surgery

Graduate School of Medical Sciences, Kyushu University

3-1-1 Maidashi, Higashi-ku, Fukuoka 812-8582, Japan

Tel: +81-92-642-5573

Fax: +81-92-642-5580

E-mail: [taguchi@pedsurg.med.kyushu-u.ac.jp](mailto:taguchi@pedsurg.med.kyushu-u.ac.jp)

***Isolation and culture of SHED and gingival fibroblasts***

Dental pulp tissues from human deciduous teeth were digested with 0.3% collagenase type I (Worthington Biochemicals, Lakewood, NJ) and 0.4% dispase II (Sanko Junyaku Co., Ltd., Tokyo, Japan) for 60 min at 37^o^C, and the obtained cells were seeded on culture flasks. After three hours, the cultures were washed with sterilized phosphate-buffered saline (PBS). The adherent cells were incubated with a regular medium containing 15% fetal bovine serum (Equitech-Bio, Kerrville, TX), 100 μM L-ascorbic acid 2-phosphate (Wako Pure Chemicals, Osaka, Japan), 2 mM L-glutamine (Nacalai Tesque, Kyoto, Japan), 100 U/ml penicillin and 100 μg/ml streptomycin (Nacalai Tesque) in alpha Modification of Eagle's Medium (Invitrogen, Carlsbad, CA). The isolated cells showed the capacity to form attached colonies consisting of spindle-shaped cells on plastic culture dishes (**data not shown**). The adherent colony-forming cells were passaged and sub-cultured. The medium was changed twice a week. To confirm whether our isolated cells were MSCs, the passage 3 (P3) cells were assessed by a flow cytometric analysis as described previously (Yamaza et al., 2010). The P3 cells were also cultured under osteogenic, chondrogenic and adipogenic conditions as described previously (Ma et al., 2012). Our definition of the present isolated cells from exfoliated tooth as MSCs was according to previous criteria (Dominici et al., 2006).

Human gingival connective tissues were treated with 0.3% collagenase type I (Worthington Biochemicals) and 0.4% dispase II (Sanko Junyaku Co., Ltd.) for 60 min at 37^o^C. The obtained cells were seeded on a culture flask, and cultured in the regular medium until a confluent condition. They were passaged and were expanded to the P3 cells.

***Transplantation of gingival fibroblasts into CCl4-treated mice***

A mixture of CCl_4_ (Wako Pure Chemicals, Osaka, Japan) (0.5 mL/kg body weight) and olive oil (Wako Pure Chemicals) (1:4 volume/volume) was intraperitoneally injected into C57BL/6J mice (male, eight weeks old) (Kyudo, Tosu, Japan) twice a week during this experimental period (**Supplementary Figure 6A**). Age- and sex-matched mice injected with olive oil were used as controls (n=5). For a control test for SHED transplantation into chronic liver disease model mice, P3 human gingival fibroblasts (1x10^6^ per mouse) suspended in 100 μl of PBS were transplanted into four-week CCl_4_-treated mice (n=5) (**Figure S6a**). As a control, 100 μl of PBS per mouse was intrasplenically infused into CCl_4_-treated mice (n=5). The mice continuously received CCl_4_ twice a week for an additional four-week treatment after the infusion. All of the animals were sacrificed to harvest the livers and peripheral blood.

***In vivo tracing of SHED***

SHED (1×10^6^) were incubated with 10 μg      , Carlsbad, CA           SHED        ^6^ per mouse into CCl4-injured mice. At 1 day after infusion, livers were fixed with 4% PFA in PBS. Frozen sections were then prepared and stained with DAPI (Dojindo).

***Histological and immunohistochemical analyses of mouse liver tissues***

Mouse organ samples were fixed overnight with 4% paraformaldehyde in PBS at 4^o^C. The samples were dehydrated, cleaned and embedded in paraffin. Six-μm-thick paraffin sections were cut. The sections were dewaxed, rehydrated, and used further staining. Masson's trichrome staining was performed as described previously (Ma et al., 2012). Some sections were also treated with hematoxylin and eosin staining. Immunohistochemical staining was performed using a DAKO EnVision kit (DAKO, Glostrup, Denmark) and primary antibodies against human leukocyte antigen-ABC (HLA-ABC) (DAKO), human hepatocyte-specific antigen hepatocyte paraffin 1 (Hep Par1) (DAKO) and human albumin (Abcam, [Cambridge](https://en.wikipedia.org/wiki/Cambridge), UK). Primary antibodies to anti-mouse alpha smooth muscle actin (Abcam), mouse F4/80 (Abcam) and mouse CD3 (Abcam). As the positive controls, human liver paraffin sections were stained with the primary antibodies. Immunohistochemical negative controls were stained with non-immune mouse and rabbit IgG instead of the primary antibodies. Finally, all of the sections were lightly counterstained with hematoxylin.

For morphometric analysis, five representative images from each mouse were randomly selected, and were used to measure the percentage of fibrous tissue area or primary antibody-positive area using the ImageJ software (NIH, Bethesda, MD).

***Double immunofluorescence***

Paraffin sections were pretreated with 10% normal goat serum and incubated with anti-human HepPar1 antibody (DAKO) followed by Alexa Fluor 594 (Life Technology, Carlsbad, CA). The sections were pretreated again with 10% normal goat serum and stained with anti-human albumin antibody (Abcam) followed by Alexa Fluor 488 (Life Technology). Finally, after washing, sections were mounted in a VECTASTAIN antifade mounting medium with 4′, 6-diamidino-2-phenylindole (DAPI) (Vector Laboratories, Burlingame, CA). The sections were observed under an Axio Imager M2 (Zeiss).

***Colorimetric analysis of mouse serum and liver samples***

The serum alkaline phosphatase (ALP) and total bilirubin were measured with a Multiskan GO microplate spectrophotometer (Thermo Scientific, Waltham, MA) using commercially available kits [ALP: LabAssay ALP Kit (Wako Pure Chemicals); total bilirubin: Bilirubin QuantiChrom Assay Kit (BioAssay Systems, Hayward, CA)] according to the manufacture’s protocol.

***Extraction of total RNAs***

Mouse liver samples were treated with TRIzol (Invitrogen), digested with DNase I (Promega, Madison, WI) and purified using an RNeasy Mini Kit (Qiagen). One microgram of purified RNA was reverse-transcribed with a Revertra Ace qPCR kit (TOYOBO, Osaka, Japan) for real-time reverse transcript-polymerase chain reaction.

***Quantitative real-time reverse transcript-polymerase chain reaction (RT-PCR) assay***

Real-time RT-PCR was subsequently performed using a TaqMan Gene Expression Master Mix (Applied Biosystems, Foster City, CA) and target TaqMan probes (Applied Biosystems) (**Table S1**) with a Light Cycler 96 (Roche, Indianapolis, IN). 18S ribosomal RNA was used for normalization.

**Supplementary References**

Yamaza T, Kentaro A, Chen C, Liu Y, Shi Y, Gronthos S, et al. [Immunomodulatory properties of stem cells from human exfoliated deciduous teeth.](http://www.ncbi.nlm.nih.gov/pubmed/20504286) Stem Cell Res Ther. 2010;1:5.

Ma L, Makino Y, Yamaza H, Akiyama K, Hoshino Y, Song G, et al. [Cryopreserved dental pulp tissues of exfoliated deciduous teeth is a feasible stem cell resource for regenerative medicine.](http://www.ncbi.nlm.nih.gov/pubmed/23251621) PLoS One. 2012;7:e51777.

Dominici M, Le Blanc K, Mueller I, Slaper-Cortenbach I, Marini F, Krause D, et al. Minimal criteria for defining multipotent mesenchymal stromal cells. The International Society for Cellular Therapy position statement. Cytotherapy 2006;8:315-317.
