# Supplementary material for: In vivo hepatogenic capacity and therapeutic potential of stem cells from human exfoliated deciduous teeth in liver fibrosis in mice
Source: Stem Cell Res Ther. 2015 Sep 10;6(1):171. doi: 10.1186/s13287-015-0154-6 (PMC4566368; doi:10.1186/s13287-015-0154-6)
Supplement: Additional file 2: — Figure S1. Showing characterization of SHED, Figure S2 showing CFSE-labeled cell tracing of donor SHED 1 day after transplantation, Figure S3 showing histological and immunohistochemical analyses of the kidney, lung, and spleen of CCl4-treated mice with splenic SHED transplantation, Figure S4 showing immunohistochemical negative control analysis, Figure S5 showing immunohistochemical analysis of human liver, Figure S6 showing transplantation of human gingival fibroblasts into CCl4-treated mice, Figure S7 showing the schema for cell sorting of human cells from recipient liver tissues of CCl4-treated mice transplanted with SHED, Figure S8 showing human HLA-ABC, hepatocyte paraffin1 (Hep Par1), or human albumin (hALB) antibody-positive area in the recipient liver tissues of secondary transplant CCL-treated mice, Figure S9 showing serum assays for the hepatic function in secondary transplant CCl4-treated mice, and Figure S10 showing fibrous assay in recipient livers of secondary transplant CCl4-treated mice. (PDF 1386 kb) [file 13287_2015_154_MOESM2_ESM.pdf]

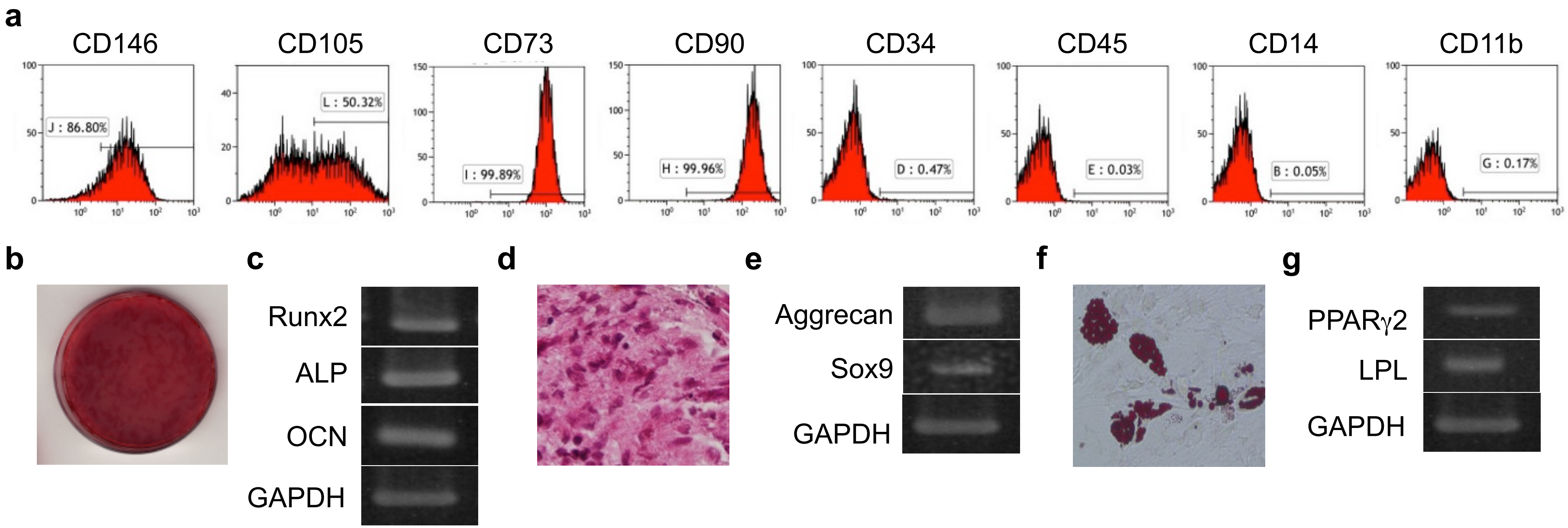

Additional file2: Figure S1. Characterization of SHED. **a** Expression of surface markers. **b** Alizarin Red staining. **c** Osteoblast/odontoblast specific gene assay. **d** Safranin O staining. **e** Chondrocyte specific gene assay. **f** Oil Red O staining. **g** Adipocyte specific gene assay.

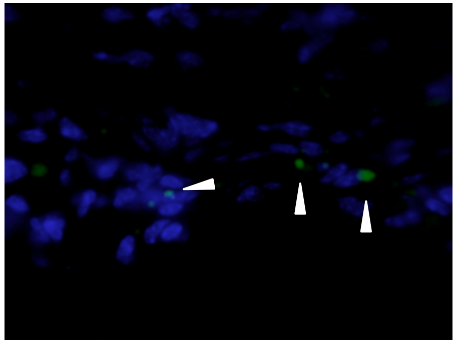

Figure S2. Tracing of CFSE-labeled donor SHED (white arrowheads) one day after the transplantation. DAPI staining.

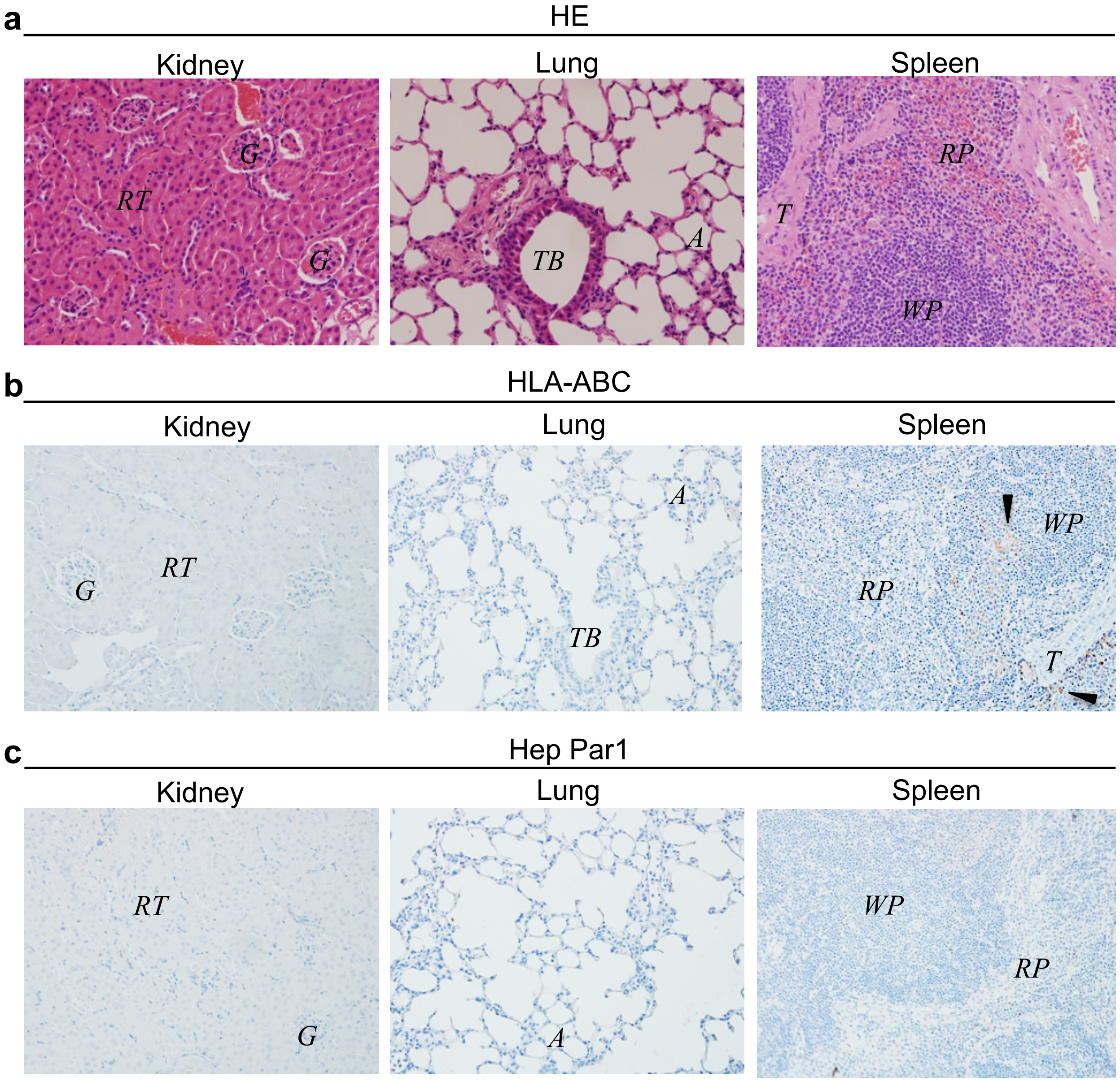

Figure S3. Histological analysis of kidney, lung and spleen of SHED transplanted recipient mice. **a** Hematoxylin and eosin (HE) staining. **b, c** Immunohistochemistry. A: alveolous, G: glomerulus, RP: red pulp, RT: renal tube, T: trabecula of spleen, TB: terminal bronchiole, WP: white pulp. Black arowheads: Immunopositive cells.

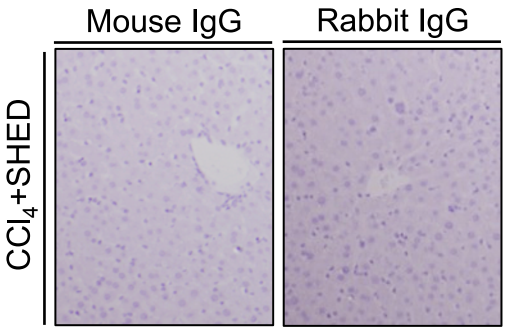

Figure S4. Immunohistochemical negative control analysis in SHED-transplanted mouse livers sections.

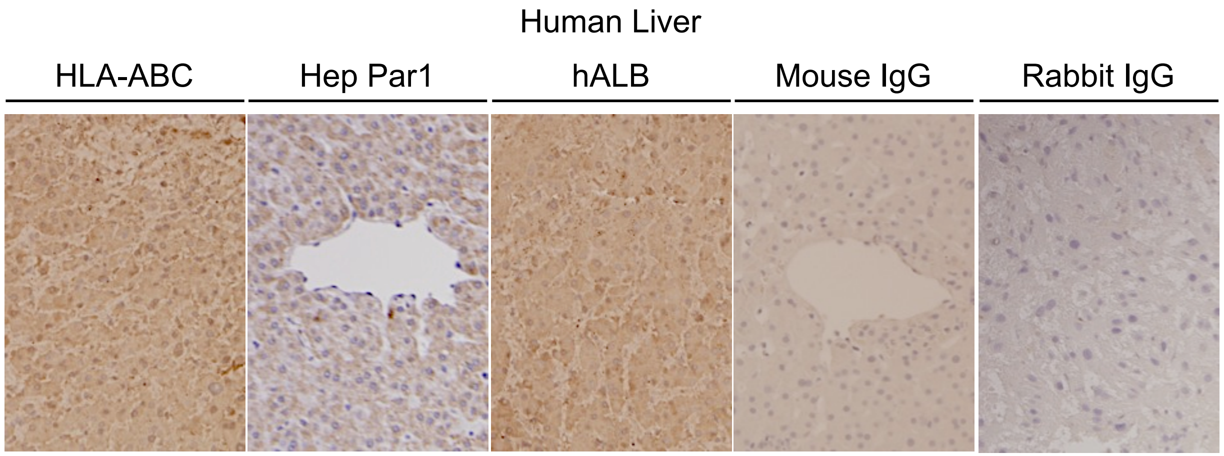

Figure S5. Immunohistochemical positive control analysis in human liver sections.

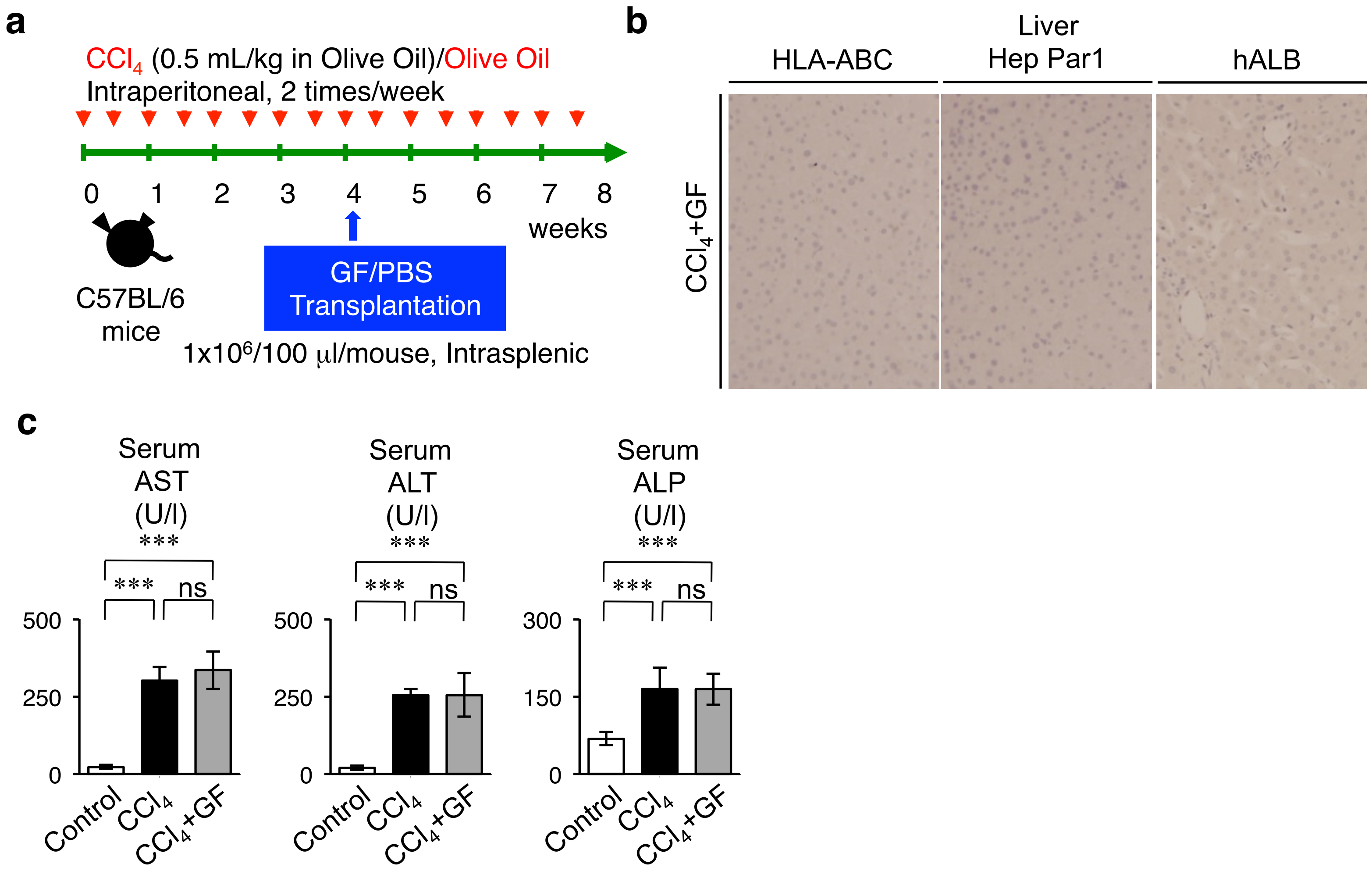

Figure S6. Transplantation of human gingival fibroblasts (GFs) into CCl_4-treated mice. **a** A schema of human GF transplantation in mice. GFs and PBS were transplanted into C57BL/6 mice received with CCl_4 or olive oil only (red arrows). **b** Immunohistochemistry. **c** Serum assays. n=5 for all groups. ****P*<0.005. ns: no significance. Graph bars show the means ± SD.

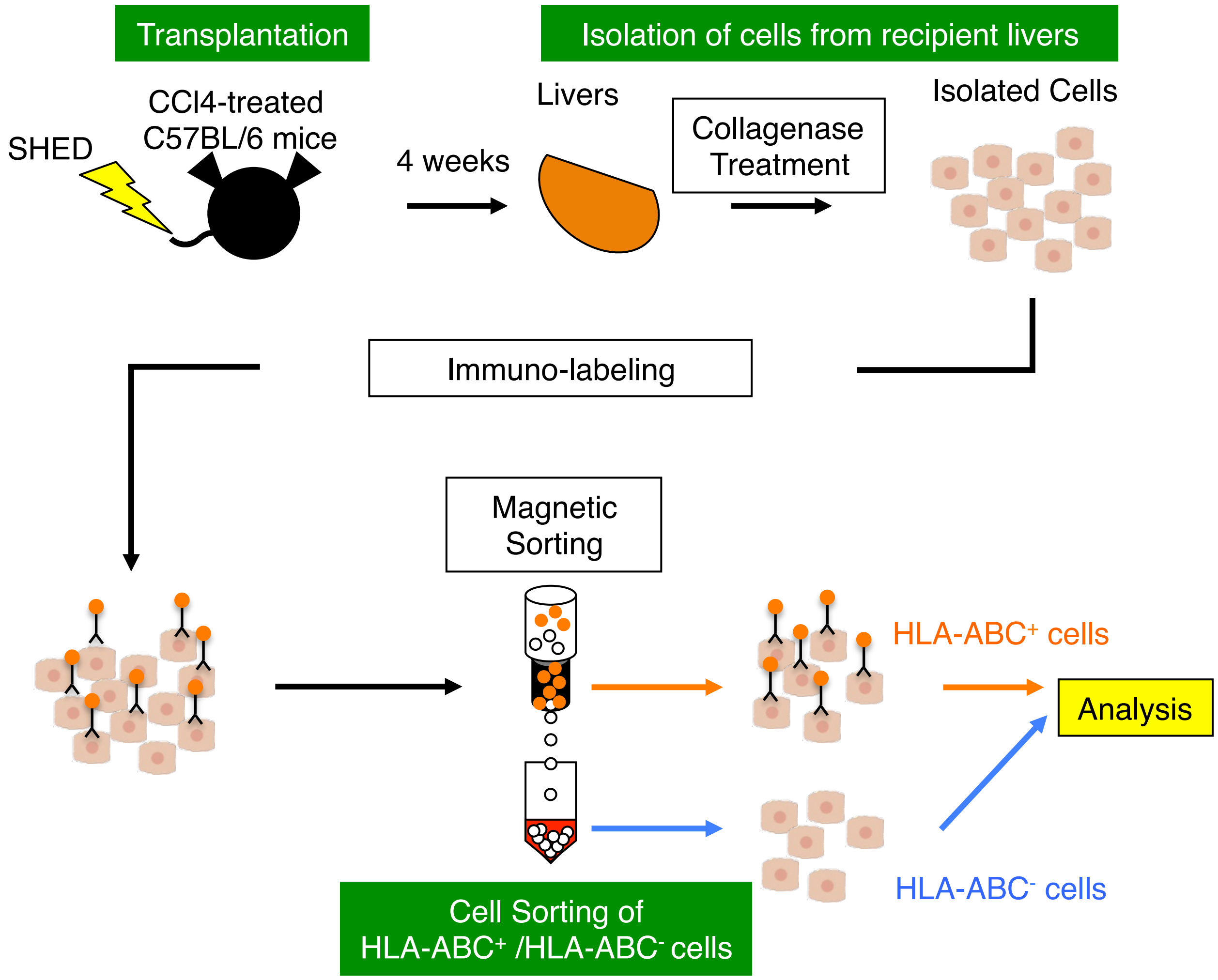

Figure S7. A schema of human cell sorting from SHED-transplanted recipient livers. Cells isolated from SHED transplanted recipient livers were immunolabeled, and magnetically sorted into HLA-ABC^{+} and HLA^{-} cells.

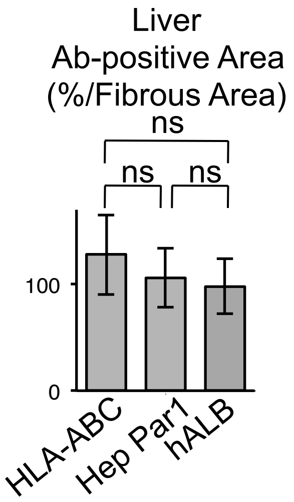

Figure S8. HLA-ABC, Hep Par1 or hALB antibody-positive area in the recipient livers of secondary transplanted mice. Immunopositive area is showed the ratio to fibrous area. n=5 for all groups.

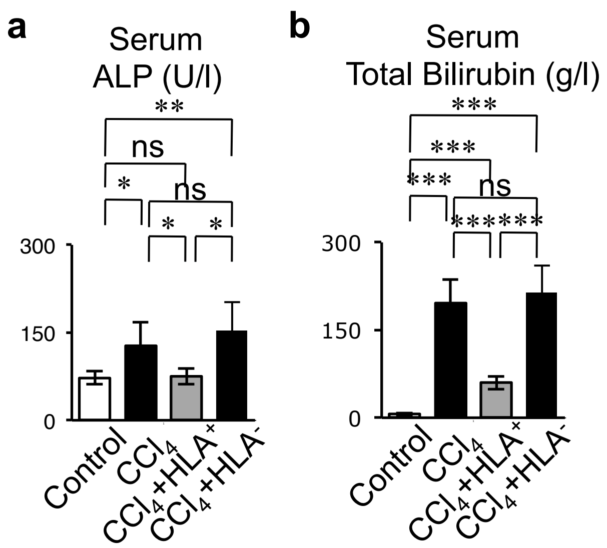

Figure S9. Serum assays for the hepatic function in secondary transplanted mice. **a** Serum ALP. **b** Serum total bilirubin. n=5 for all groups. **P*<0.05 and ***P*<0.01.

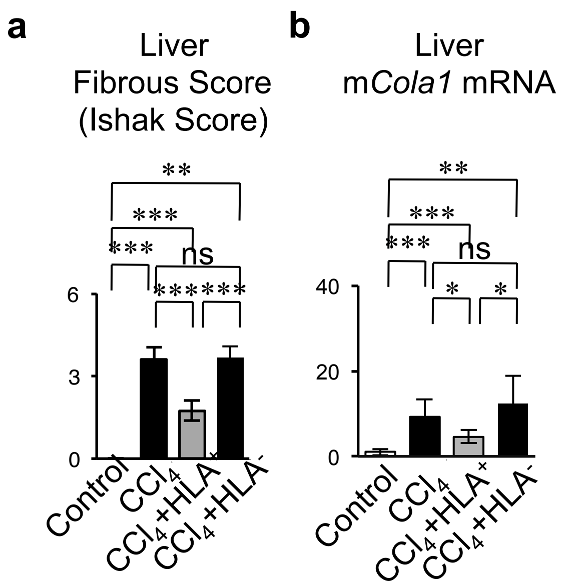

Figure S10. Fibrous assay in recipient livers of secondary transplanted mice. **a** Ishak fibrous score. **b** Expression of mouse type I collagen mRNA. n=5 for all groups.
