# Supplementary material for: In vivo hepatogenic capacity and therapeutic potential of stem cells from human exfoliated deciduous teeth in liver fibrosis in mice
Source: Stem Cell Res Ther. 2015 Sep 10;6(1):171. doi: 10.1186/s13287-015-0154-6 (PMC4566368; doi:10.1186/s13287-015-0154-6)
Supplement: Additional file 3: — Table S1. Presenting the TaqMan primers and probes used for mouse genes used in real-time PCR, and Table S2 presenting the primer pairs used for human and mouse genes for genomic PCR and RT-PCR. (DOC 65 kb) [file 13287_2015_154_MOESM3_ESM.doc]

**Additional file 3: Table S1. The TaqMan primers and probes used for mouse genes used in real-time PCR**

| *Gene names* | *Gene assay ID numbers* |
| --- | --- |
| interleukin 6 | Mm00446190_m1 |
| matrix metalloproteinase 2 | Mm00439506_m1 |
| matrix metalloproteinase 9 | Mm00600164_m1 |
| collagen type I α1 | Mm01546133_m1 |
| smooth muscle actin, alpha | Mm01341361_m1 |
| tissue inhibitor of metalloproteinase 1 | Mm00441825_m1 |
| tissue inhibitor of metalloproteinase 2 | Mm00441825_m1 |
| transforming growth factor beta 1 | Mm00443258_m1 |
| tumor necrosis factor alpha | Mm00443258_m1 |
| 18S ribosomal RNA | Mm03928990_g1 |

**Additional file 3: Table S2. The primer pairs used for human and mouse genes for genomic PCR and RT-PCR**

| ***Gene names*** | ***Forward primer sequences (5’-3’)*** | ***Reverse primer sequences (5’-3’)*** | ***GeneBank accession No*** |
| --- | --- | --- | --- |
| albumin | ATGGATGATTTCGCAGCTTT | TGGCTTTACACCAACGA | NM_000477.5 |
| Alu | CGAGGCGGGTGGATCATGAGGT | TCTGTCGCCCAGGCCGGACT | X53550 |
| coagulation factor VIII, procoagulant component | TTTTCCCCTCCTGGGAGCTAAAGAT | ACCCATCCAGGGTGGCCTTG | NM_000132.3 |
| cytochrome P450, family 1, subfamily A, polypeptide 1 | CCCAGGCTCCAAGAGTCCACCC | AATCATCGCCCTGCCGCACC | NM_000499.3 |
| cytochrome P450, family 3, subfamily A, polypeptide 7 | TCCTTCCGTAAGGGCTATTGGACG | GCCTGTCTCTGCTTCCCGCC | NM_000765.3 |
| fumarylacetoacetate hydrolase | GCACCCCAATCCGAAGGCCC | ATGGGCACCACCCACGGAGA | NM_000137.2 |
| glyceraldehyde-3-phosphate dehydrogenase | TGGCGCTGAGTACGTCGTGG | ACGTTGGCAGTGGGGACACG | NM_002046.3 |
| mpf1 | CCGGGCAGTGGTGGCGCATGCCTTTAAATCCC | GTTTGGTTTTTGAGCAGGGTTCTCTGTGTAGC | X78319 |
| transferrin | CGGCAGTTGGCAGAACCGCT | CCACAGCGTGATTCGGGGCT | NM_001063.3 |
| transthyretin | TGCCATCAATGTGGCCGTGC | GCAATGGTGTAGCGGCGGGG | NM_000371.3 |
| tyrosine aminotransferase | CCGGGAGAGTTTTACCACAA | CCTTCCCTAGATGGGACACA | NM_000353.2 |
| UDP glucuronosyltransferase 1 family, polypeptide A1 | TGAGGCTACCCAGTGCCCCAA | GTCGGGTTCCAGTGTACCGCC | NM_000463.2 |
